# The Utility of Psoas Muscle Assessment in Predicting Frailty in Patients Undergoing Transcatheter Aortic Valve Replacement

**DOI:** 10.1155/2020/5783107

**Published:** 2020-06-28

**Authors:** Louis Koizia, Mitesh Naik, George Peck, Ghada W. Mikhail, Sayan Sen, Iqbal S. Malik, Ben Ariff, Michael B. Fertleman

**Affiliations:** ^1^Cutrale Perioperative and Ageing Group, Imperial College, London, UK; ^2^Department of Radiology, Imperial College Healthcare NHS Trust, London, UK; ^3^Department of Geriatrics, Imperial College Healthcare NHS Trust, London, UK; ^4^Department of Cardiology, Imperial College Healthcare NHS Trust, London, UK

## Abstract

**Background:**

The rise in an ageing population has resulted in an increase in the prevalence of aortic stenosis. With the advent and rapid expansion in the use of transcatheter aortic valve replacements (TAVRs), patients with severe aortic stenosis, traditionally thought too high risk for surgical intervention, are now being treated with generally favourable results. Frailty is an important factor in determining outcome after a TAVR, and an assessment of frailty is fundamental in the identification of appropriate patients to treat.

**Objective:**

The objective of the study was to identify if the psoas muscle area is associated with frailty in TAVR patients and outcome after intervention.

**Method:**

In this prospective study, we measured outcomes of 62 patients who underwent TAVR procedures against the psoas muscle area and the Reported Edmonton Frail Scale (REFS). Our aim was to assess if psoas muscle assessment can be used as a simple method to predict frailty in our population group.

**Results:**

A total of 60 patients met the study criteria. Mean psoas-lumbar vertebral index was 0.61, with a lower value in the frail group. There was not a statistically significant correlation between the psoas measures, REFS score (indicative of frailty), and mortality. However, there was a statistically significant relationship between the psoas size and REFS score (*p*=0.043).

**Conclusion:**

Psoas assessment can be useful in providing additional information when planning for patients to undergo a TAVR and can be used as a screening tool to help identify frail patients within this high-risk group.

## 1. Introduction

Severe aortic stenosis is associated with poor prognosis and significant morbidity, particularly, in patients with concomitant left ventricular failure. Transcatheter aortic valve replacement (TAVR) has revolutionized the treatment of patients with aortic stenosis (AS) over the last 15 years [1], and there is now evidence to suggest TAVR should be the preferred approach across the spectrum of surgical risk, with the 2019 multicenter PARTNER 3 study showing lower rates of death, stroke, or rehospitalization at 1 year with a TAVR compared with a surgical aortic valve replacement (SAVR) [2]. The U.S. Food and Drug Administration (FDA) issued its approval of TAVR in low surgical risk patients, becoming the first regulatory body in the world to do so [3].

Despite this, there is yet to be an updated guideline for the management of AS. Current European Society of Cardiology (ESC) and European Association for Cardio-Thoracic Surgery (EACTS) 2017 guidelines for AS management recommend consideration for TAVR in patients with severe symptomatic AS who are deemed to be too high risk for cardiac surgery [4]. The guidelines recommend that TAVR decisions are taken by the “Heart Team,” including cardiologists, cardiothoracic surgeons, and anaesthetists. Multidisciplinary teams (MDTs) can make use of risk scores to support decision making. Commonly used risk scores including Society of Thoracic Surgeons (STS) and EuroSCORE II have been found to be inaccurate at predicting mortality and morbidity in TAVR patients [5, 6]. This is felt to be related to the complexity of this subgroup of patients, who are frequently frail and have multiple comorbidities [7].

### 1.1. Frailty and Sarcopenia in TAVR

Frailty is a recognized clinical entity, independent of age, comorbidity, and disability. It is defined as a state of reduced physiological reserve and associated with an increased susceptibility to poor healthcare outcomes [8]. Frailty has been shown to result in worse postoperative recovery across surgical specialties [9]. Green et al. identified increased mortality and higher rates of poor outcomes at one year following a TAVR, in frail patients [10]. Kiani et al. identified a relationship between the indices of frailty (anaemia, albumin, and 5 metre walk speed), with length of stay, rates of bleeding, and readmission [11]. Other studies have shown similar relationships, but use complex frailty scores or difficult to perform tests in clinical practice [12, 13]. For example, Huded et al. used a modified Fried frailty assessment that comprised four domains and required specialist equipment [14]. Schoenenberger et al. described a significantly enhanced prediction of 1-year mortality when combining multifactorial frailty indices, which may be onerous and time-consuming, with aforementioned STS and EuroSCORE risk scores [12]. The multicentre FRAILTY-AVR study recommended the abbreviated Essential Frailty Toolset (EFT), a 4-item scale encompassing lower-limb weakness, cognitive impairment, anaemia, and hypoalbuminaemia, to assess frailty before TAVR or SAVR, but again, a documented limitation of this is its time-consuming process [15].

The Edmonton Frail Scale (EFS) is a frailty assessment that comprises 10 questions and one physical assessment (“timed up and go”). The EFS has been validated against the Comprehensive Geriatric Assessment (CGA), the current gold-standard for frailty assessment, and has been shown to be reliable and feasible for routine use by non-geriatricians [16]. Scores range from 0 (not frail) to 18 (very frail), with scores of 8 or above being defined as frail. Dasgupta et al. described the use of Edmonton Frail Scale (EFS) on patients pre-operatively, in advance of elective orthopaedic operations. This study found that individuals with a score of 7 or more were likely to have greater postoperative complications and less likely to be discharged home [17]. The Reported Edmonton Frail Score (REFS) is an adaptation of EFS; substituting the last domain on EFS, the physical performance measure, with three self-assessed physical performance questions (Table 1) [18]. It is common for exercise tolerance to be affected by worsening AS, and thus, patients may not perform as well in the physical assessment part of the EFS [19].

Sarcopenia is defined as a reduction in the muscle mass, contributing to decreased strength and low physical performance [20]. It is considered a key component of the “frailty syndrome” with the proposed pathophysiology including reduced capillary blood flow, mitochondrial abnormality, anorexia, neuronal loss, and insulin resistance, and has been found to be associated with poor outcome in older people after critical illness, surgery, and traumatic injury [21–24]. The psoas muscle has been shown as a reliable indicator of sarcopenia and a useful surrogate marker for frailty, being simple and convenient to measure [25].

Skeletal muscle area at L4-5 has been shown to correlate with total body muscle volume making this a useful level for the assessment of the overall muscle mass [26]. To adjust for body size, height is sometimes used for correction to create a “skeletal muscle index,” but this is a measure often unavailable in clinical practice [27]. An alternative to using height to correct for the body size is by using vertebral body size, which is advantageous as this is acquired on CT together with the psoas size. The “psoas-lumbar vertebral index” (PLVI) has been associated with poor outcomes in older trauma, acetabular fractures, kyphoplasty, and in patients undergoing knee or hip arthroplasty [28–31].

Psoas assessment is ideal for use in TAVR patients, given that routine workup includes a CT angiogram study, which covers the psoas within the imaged volume. Psoas sarcopenia has been identified as a strong predictor of long term mortality after TAVR [32–35].

The purpose of this study was to evaluate the correlation between psoas assessment and a validated frailty score (REFS). Secondary outcomes were to identify 30-day and 18-month mortality.

## 2. Methods

### 2.1. Study Population

Consecutive patients with severe symptomatic aortic stenosis referred for evaluation at Imperial College Healthcare NHS Trust, considered high risk for surgical aortic valve replacement (SAVR), but eligible for TAVR, were included. This group of high-risk patients was assessed in the TAVR clinic. Following the clinic encounter, each patient was discussed in an MDT that consisted of interventional cardiologists, cardiothoracic surgeons, an anaesthetist, radiologists, and a geriatrician. A consensus was reached amongst the MDT about offering a TAVR. Patients were excluded if they had TAVR as an emergency or they were not reviewed by the geriatrician undertaking the REFS prior to their procedure. The REFS was performed with the patient and/or caregiver. Patients were followed up for 18 months or until the date of death.

#### 2.1.1. Cardiac Data

All patients had extensive cardiac baseline examinations including echocardiography to evaluate the left ventricular ejection fraction, aortic valve orifice area and mean gradient, in addition to coronary angiography, CT angiography, and lung function tests. Symptomatic history was elicited including allocation to the NYHA classification.

#### 2.1.2. Psoas Assessment

**Figure 1:**
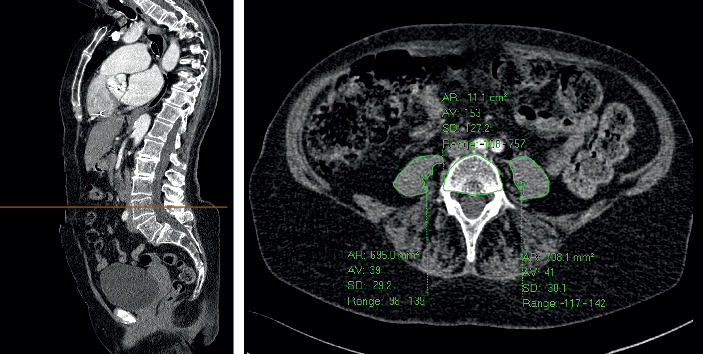
Cross-sectional psoas and vertebral body areas derived from an axial CT image at the level of L4, using a freehand region-of-interest tool (green outlines). In this patient, the PLVI was mean psoas area (mm^2^)/vertebral body area (mm^2^) ((695 + 708)/2)/1110 = 0.63.

All patients underwent a CT angiogram as part of their imaging workup prior to TAVR. An independent trained radiologist visually analyzed these CT studies retrospectively. Axial sections at the level of the fourth lumbar (L4) vertebral body, immediately inferior to the origin of the posterior elements, were taken, the point at which the cross-sectional area of the left and right psoas muscles was measured. This was carried out using a pre-existing freehand region of interest tool available on the Carestream Picture Archive and Communications System. The mean psoas cross-sectional area was calculated. To correct for body habitus, a record was also made of the cross-sectional area of the L4 vertebral body at the same level. From these data, the mean total psoas cross-sectional area was divided by the L4 vertebral body cross-sectional area to calculate PLVI (Figure 1).

#### 2.1.3. TAVR Procedure

A Medtronic CoreValve or an Edwards Sapien XT bio-prosthesis was implanted. The transcatheter aortic valve was introduced transfemorally whenever feasible; otherwise, subclavian routes were adopted.

#### 2.1.4. Statistics

The chi-square test was used for the assessment of two categorical variables, and the Mann–Whitney test was used for nonparametric variables. For all statistical analyses, we used commercially available software (GraphPad QuickCalcs Software).

## 3. Results

### 3.1. Study Population

Frailty assessment was performed on 62 patients with severe symptomatic aortic stenosis who subsequently underwent TAVR. The mean age was 84 years (range: 68 to 95) with 26 being females (42%).

Of the original 62 patients to be included, 60 had psoas measurements performed (two excluded due to poor image quality or CT scan being performed at a different hospital). The mean psoas-lumbar vertebral index was 0.61 with characteristics described in Table 2.

### 3.2. Mortality

Three (5%) patients died within 30 days of undergoing TAVR. At 18 months, 10 (16%) all-cause deaths were noted (Table 2).

### 3.3. Psoas-Lumbar Vertebral Index and the Frailty Score

Participants were categorized as non-frail (a REFS less than 8) or frail (a REFS more than or equal to 8). The PLVI was compared between the two groups (Table 3). Frail patients had a lower PLVI than non-frail patients. (the Mann–Whitney *U z*-score 1.67, *p* = 0.047). The lowest quartile PLVI patients were defined as sarcopenic, as described in prior studies [36]. Sarcopenic vs. non-sarcopenic groups were compared with REFS; (frail or non-frail) (Table 4) using the chi-squared test which demonstrated a significant association between high PLVI and the non-frail status (4.09) (*p* = 0.043 confidence interval 95%).

## 4. Discussion

The association between frailty and poor health outcome is well-documented in the literature, and there is emerging evidence showing a similar association between frailty and outcome following TAVR. Relatively limited information is available relating to the use of psoas assessment as a predictor of frailty in TAVR patients [8] compared with other indices. In this study, we aimed to investigate if the psoas area can be a simple and straightforward method of predicting frailty in TAVR patients. In addition, we wanted to identify the utility of using psoas measurements to predict mortality and the length of stay following TAVR.

Despite the patients in our cohort being classified as “high risk,” the vast majority (95%) survived to discharge and were discharged to their original place of residence. In particular, 84% (52) patients were still alive at 18 months after TAVR, despite being deemed too high risk for SAVR. This compares favourably to the outcomes observed in the original TAVR trials.

Previous studies have evaluated the use of complex frailty assessments to identify frail patients undergoing TAVR and identified a correlation between all-cause mortality and frailty [37]. However, the assessments used can be difficult to adopt in clinical practice, proving burdensome and time-consuming for both the clinician and the patient, and they can be subject to variability depending on the patient performance at the time of assessment. In contrast, assessment of psoas muscle using pre-operative CT is feasible, quick, and relatively straightforward. Our study identifies that the use of the psoas-lumbar vertebral index is significantly associated with the REFS, a validated method for predicting frailty. Therefore, the calculation of the index can be utilized as a method to predict or augment frailty assessment amongst a group with likely multiple comorbidities. The advantages of using the PLVI are that it is reproducible, quantitative, and not affected by day-to-day fluctuations in the functional state. It adjusts for the body size without the need for a recorded height and is simple to collect, only adding a few minutes to a radiologist's reporting time during the interpretation of a scan which is already performed for other reasons. Our results suggest that a PLVI of less than 0.52 identifies frail TAVR patients.

Although several studies have now described the relationship between psoas sarcopenia and poor outcomes following TAVR, this association between psoas muscle and the outcome after TAVR is not straightforward [38–40]. Mamane et al. identified a correlation between the psoas muscle area and 6-month mortality in female patients only [33] in a relatively small cohort, and another study by van Mourik et al. showed similar findings [39]. The use of sarcopenia of the psoas muscles as a surrogate for overall frailty has also been well-described in other patient groups [41–43], but there is some conflicting evidence regarding whether the psoas area is truly representative of the global skeletal muscle area [44]. Its routine use, particularly, in research studies, is, in part, secondary to convenience and simplicity of acquiring measurements, but it is suggested in the literature that the total lumbar muscle area may be more closely related to the overall muscle mass, and given that psoas comprises only 10% of the total trunk musculature, this may not be accurate [45, 46]. Indeed, Krishnan et al. reported that sarcopenia was underestimated by 10% using psoas muscles only compared with paravertebral muscles on CT imaging in a cohort of patients who underwent TAVR [40]. The prospect of muscle segmentation on volumetric CT imaging using deep learning algorithms provides an exciting opportunity for work in this area and may streamline the collection of important quantitative measures to improve reproducibility [47, 48]. Vertebral body fractures or other vertebral anomalies may affect the reliability of the PLVI given use of the adjacent bone to correct for the body size. This method was used in our study as patient heights were not available, although correction using height has been described more frequently in the previous literature. Although there is a paucity of data surrounding the difference between correcting using the vertebral body size compared with the height, this may have impacted our results.

Further collaborative work is required with larger samples sizes to compare both overall results of different frailty scores and their clinical feasibility, to determine which of the plethora of frailty scales offers most overall prognostic benefit [15]. This is even more pertinent, given the ageing population and a likely increase in frailty in patients undergoing interventional procedures. In addition, given the expanding role of TAVR in clinical practice and inevitable rollout amongst low- and intermediate-risk patient groups, combining frailty indices with objective measures of sarcopenia may be the most predictive technique rather than either of these tools alone, and this warrants ongoing prospective investigation [49].

### 4.1. Limitations

The lack of a significant relationship between outcomes after TAVR and the psoas muscle area in our study can be explained by the small sample size from a single centre. The retrospective nature of the study meant that analysis was restricted by missing data; it was not possible to assess further aspects of sarcopenia such as muscle strength and performance or to acquire patients' heights to use this to correct the psoas area.

In addition, the study did not follow up or further assess patients who did not undergo intervention and received medical management alone. It is likely that this group would have the highest mortality and most likely to be sarcopenic.

## 5. Conclusions

PLVI demonstrates an effective, simple, and relatively quick method for predicting sarcopenia and frailty in TAVR patients, who will routinely undergo a preprocedure CT scan as part of their workup. Our study identifies a relationship between the PLVI and a validated frailty score (REFS). PLVI may be a useful adjunct in identifying patients who are at the most risk of prolonged admission or poor outcome following TAVR. Further research is required to assess the utility of psoas assessment in determining outcomes in TAVR patients.

## Figures and Tables

**Table 1:** Reported Edmonton Frail Scale, adapted from Hilmer et al. [18].

Domain	Item	0 point	1 point	2 points
Cognition	Predrawn circle. Add the numbers in the correct positions to make a clock then place the hands to indicate a time of ten after eleven	No errors	Minor errors	Major errors
General health	In the past year, how many times have you been admitted to a hospital?	0	1-2	>2
	In general, how would you describe your health?	Good/ excellent	Fair	Poor
Functional independence	With how many of the following activities do you require help? (i) meal preparation (ii) shopping (iii) transportation (iv) telephone (v) housekeeping (vi) laundry (vii) managing money (viii) taking medications	0-1	2–4	>4
Social support	When you need help, can you count on someone who is willing and able to meet your needs?	Always	Sometimes	Never
Medication use	Are you on five or more different prescription medications on a regular basis?	No	Yes	
	At times, do you forget to take your prescription medications?	No	Yes	
Nutrition	Have you recently lost weight such that your clothing has become looser?	No	Yes	
Mood	Do you often feel sad or depressed?	No	Yes	
Continence	Do you have a problem with losing control of urine when you do not want to?	No	Yes	
Functional Performance	Two weeks ago, were you able to:			
	Do heavy work around the house like washing windows, walls, or floors without help?	Yes	No	
	Walk up and down stairs to the second floor without help?	Yes	No	
	Walk 1 km without help?	Yes	No	

**Table 2:** Characteristics of cohort in quartiles of the psoas-lumbar vertebral index including mortality and the length of stay.

	1^st^ quartile	2^nd^ quartile	3^rd^ quartile	4^th^ quartile
Female (%)	53	60	47	7
Age	84	85	85	81
Creatinine	91	88	105	121
Body mass index	23.5	26.9	25.1	31.7
30-day mortality	1	1	0	1
18-month mortality	3	1	0	5
Discharged home (%)	86	100	93	93
Length of stay (days)	5.07	5.64	5.93	9.86

**Table 3:** The mean, median, and range of the psoas-lumbar vertebral index amongst the frail and non-frail participants (*p*=0.047).

	*N*	Mean	Median	Range
Non-frail	45	0.63	0.62	0.29–0.95
Frail	15	0.54	0.56	0.20–0.72

**Table 4:** Comparing the psoas-lumbar vertebral index and REFS scores. A significant relationship was found between high PLVI and the non-frail status (*p*=0.043).

	Frail as per REFS (score > 7)	Non-frail (score < 8)
Sarcopaenia (PLVI < 0.52)	7	8
Non-sarcopaenia (PLVI ≥ 0.52)	9	36

## Data Availability

The data used to support the findings of this study are available from the corresponding author upon request.
